# Supplementary material for: Manipulating surface magnetic order in iron telluride
Source: Sci Adv. 2019 Mar 1;5(3):eaav3478. doi: 10.1126/sciadv.aav3478 (PMC6397027; doi:10.1126/sciadv.aav3478)
Supplement: http://advances.sciencemag.org/cgi/content/full/5/3/eaav3478/DC1 [file supp_5_3_eaav3478__index.html]

Science Advances | Science Advances

## Supplementary Materials

**This PDF file includes:**

- Section S1. DFT calculation of magnetic contrast
- Section S2. SP-STM study of the magnetic structure of Fe1.06Te
- Section S3. Incommensurate order in Fe1.16Te
- Section S4. Manipulating the surface excess Fe concentration
- Section S5. Alternative method to determine sample spin polarization
- Section S6. Model for the magnetic structure at *x* = 0.12
- Section S7. Model for the magnetic structure at *x* = 0.2
- Fig. S1. Spin-polarized imaging at low excess iron concentrations *x* < 0.12.
- Fig. S2. Spin-polarized imaging at high excess iron concentrations *x* > 0.12.
- Fig. S3. Manipulation of surface excess iron with aggressive tunneling parameters.
- Fig. S4. Manipulation of surface excess iron with moderate tunneling parameters.
- Fig. S5. Manipulating surface magnetic order.
- Fig. S6. Extracting surface spin polarization.
- Fig. S7. Simulated SP-STM images for *x* = 0.12.
- Table S1. Crystal structure of Fe1+*x*Te at different excess iron concentrations *x*.
- References (*32*–*37*)

Download PDF

**Files in this Data Supplement:**

- Adobe PDF - aav3478\_SM.pdf
