# Supplementary material for: Manipulating surface magnetic order in iron telluride
Source: Sci Adv. 2019 Mar 1;5(3):eaav3478. doi: 10.1126/sciadv.aav3478 (PMC6397027; doi:10.1126/sciadv.aav3478)
Supplement: Download PDF [file aav3478_SM.pdf]

Science Advances
AAAS

advances.sciencemag.org/cgi/content/full/5/3/eaav3478/DC1

Supplementary Materials for

Manipulating surface magnetic order in iron telluride

Christopher Trainer, Chi M. Yim, Christoph Heil, Feliciano Giustino, Dorina Croitori, Vladimir Tsurkan, Alois Loidl, Efrain E. Rodriguez, Chris Stock, Peter Wahl*

*Corresponding author. Email: wahl@st-andrews.ac.uk

Published 1 March 2019, *Sci. Adv.* **5**, eaav3478 (2019)
DOI: 10.1126/sciadv.aav3478

This PDF file includes:

Section S1. DFT calculation of magnetic contrast
Section S2. SP-STM study of the magnetic structure of $Fe_{1.06}Te$
Section S3. Incommensurate order in $Fe_{1.16}Te$
Section S4. Manipulating the surface excess Fe concentration
Section S5. Alternative method to determine sample spin polarization
Section S6. Model for the magnetic structure at $x = 0.12$
Section S7. Model for the magnetic structure at $x = 0.2$
Fig. S1. Spin-polarized imaging at low excess iron concentrations $x < 0.12$.
Fig. S2. Spin-polarized imaging at high excess iron concentrations $x > 0.12$.
Fig. S3. Manipulation of surface excess iron with aggressive tunneling parameters.
Fig. S4. Manipulation of surface excess iron with moderate tunneling parameters.
Fig. S5. Manipulating surface magnetic order.
Fig. S6. Extracting surface spin polarization.
Fig. S7. Simulated SP-STM images for $x = 0.12$.
Table S1. Crystal structure of $Fe_{1+x}Te$ at different excess iron concentrations x.
References (*32–37*)

Section S1. DFT calculation of magnetic contrast

The calculations were performed using the density functional theory in the generalized gradient approximation (*32*), as implemented in the QUANTUM ESPRESSO package (*33*). To describe the bicollinear magnetic order of FeTe, a $2\times1\times1$ supercell is considered, with details of the crystal structure and atomic positions taken from experiment (*34*). The magnetic moment changes upon structural relaxation (20%), however the STM images remain essentially the same when using the experimental structure or the optimized structure. We used optimized norm-conserving Vanderbilt pseudopotentials (*35*) with semi-core states included for both Fe and Te and we counter-checked our results using projector-augmented plane wave pseudopotentials (*36*). A kinetic energy cutoff of 100 Ry was chosen for the wavefunctions. The electronic Brillouin zone of the magnetic supercell was sampled using $12\times24\times12$ points. To simulate STM maps, a vacuum region of 10Å was added to 2 FeTe layers. Including more FeTe layers did not have any effects on our results.

The tunneling current in an STM experiment at small bias voltages can be approximated by (*37*)

$$I_{STM} = \text{const} \cdot \sum_{j:E_{S,j}^{\uparrow/\downarrow}>E_F}^{E_F+|V_{bias}|} \left(|\psi_j^{\uparrow}(\mathbf{r})|^2 \pm |\psi_j^{\downarrow}(\mathbf{r})|^2 \right)$$

where E_F is the Fermi energy of the sample, E_j the energy of the electron wavefunction $\psi_j(\mathbf{r})$ of the sample at the tip position $\mathbf{r}$ and the arrows indicate the two different spin polarizations. The plus and minus signs in this expression are for spin-unpolarized and spin-polarized calculations, respectively, corresponding to STM measurements using a non-magnetic tip and maps of the spin-polarization obtained by subtracting two SP-STM images obtained with oppositely polarized tips from each other. Simulated (SP-)STM images are calculated for an average tip-sample distance of 5Å and bias voltage of 0.1V.

Section S2. SP-STM study of the magnetic structure of $Fe_{1.06}Te$

Here we summarize SP-STM measurements carried out on multiple samples of $\mathrm{Fe}_{1+x}\mathrm{Te}$ with low excess Fe concentration x. On each sample that we have measured only single q magnetic order with a wave vector $\mathbf{q} = (0.5, 0)$ was ever observed. To determine the magnetic structure we have measured the surface spin polarization on multiple samples using ferromagnetic STM tips. By repeating this process with three orthogonal field directions we have acquired information about the full 3D magnetic structure at the surface of the sample [see fig. S1(a-c)]. For this procedure to produce valid data the atomic structure of the tip apex must not change throughout the measurement. From these data, we can extract spatial maps of the in-plane [fig. S1(d)] and out-of-plane [fig. S1(e)] angle of the spins with respect to the crystallographic b direction. In every sample that we have studied we see a sizable out-of-plane tilting of the magnetization away from the FeTe plane. We determine the surface magnetization to be tilted by $28 \pm 3°$ with respect to the FeTe plane on average [see fig. S1(f)]. We also detect a small tilting of $\sim 7\,°$ of the in-plane component of the magnetization away from the crystal b axis.

Section S3. Incommensurate order in $Fe_{1.16}Te$

To determine the wavelength of the magnetic ordering in samples of $\mathrm{Fe}_{1+x}\mathrm{Te}$ on the orthorhombic side of the phase transition, we have measured the spin polarization of a sample of FeTe with an excess Fe concentration of 16%. To this end, we have imaged the sample surface without removing the surface Fe to preserve as closely as possible the conditions as in the bulk of the materials as probed by neutron scattering. We extract the spin polarization by taking the difference of two images recorded with oppositely polarized tips. From the Fourier transformation of the difference image we can extract the q-vector of the magnetic order (see fig. S2) From this procedure we find an incommensurate wave vector of 0.39, in excellent agreement with neutron scattering. (*2*)

The quality and magnetic properties of STM tips after manipulation have been judged from atomic resolution images and spin-polarized STM images taken in different magnetic fields. All results presented in this work have been confirmed with different micro tips (i.e. tips with a different apex).

Section S4. Manipulating the surface excess Fe concentration

To study the effect of the interstitial Fe atoms on the magnetic order of $\mathrm{Fe}_{1+x}\mathrm{Te}$ we have used the STM tip to remove them from the surface. This was done in two ways: (1) the surface can be cleaned by using aggressive tunneling parameters while scanning the STM tip and (2) the Fe atoms can be more gently removed to study how the removal of the Fe atoms affects the magnetic order and how the tip collects the Fe. For the first method, the tip sample tunneling parameters are typically set to $I_t = 6\mathrm{nA}$ and $V_t = 500\mathrm{mV}$ and the tip is quickly scanned across the surface with a minimal feedback response time. This allows for the complete removal of almost all of the surface Fe atoms as can be seen in fig. S3. The cleaned areas on the high excess Fe samples that were studied in the main text were selected from larger areas that had been cleaned by this method. Secondly it is possible to see exactly how the excess Fe is removed by the tip. This is done by employing slightly less aggressive tunneling parameters than those used to completely remove all of the surface interstitial Fe. Typically bias voltages in the range of $30\mathrm{mV}$ to $150\mathrm{mV}$ are used with a tunneling current between 1 and $2\mathrm{nA}$. The surface is then repeatedly scanned with these parameters which allow for the observation of how the Fe atoms are removed. It can be seen from the sequential images shown in fig. S4(a) to (c) that the Fe atoms are dislodged from the surface and group together to form clusters, it is then these clusters that are collected by the tip. By repeatedly scanning an area on a sample with a 12% excess Fe concentration it is then possible to see how the magnetic order changes as the Fe is dislodged from its position and removed. Figure S4(d) and (e) show the same area imaged

before and after manipulation of the Fe atoms. As the Fe is dislodged and moved between (d) and (e) it is possible to see the spread of the bicollinear magnetic order to areas that previously had weak magnetic ordering and a high excess Fe concentration.

Figure S5 shows the same data as in Fig. 3 of the main manuscript, showing the Fourier transformations of the regions marked by a blue and green box, as well as a line cut through the peak due to the magnetic order, highlighting that excess iron rich regions show dominantly an incommensurate wave vector of the magnetic order, whereas regions where the excess iron has been removed exhibit the bicollinear order with a commensurate wave vector. We note that even in the excess iron rich region, a weak component of the magnetic order is seen at the commensurate wave vector, which we attribute to areas with locally lower excess iron concentration.

Section S5. Alternative method to determine sample spin polarization

The equation that represents spin polarized tunneling in an STM setup is given by $I = I_0(1 + P_{\text{tip}}P_{\text{sample}}\cos\theta)$, where I_0 is the tunneling current that would be measured with a non magnetic tip, P_{tip} is the spin polarization of the tip and P_{sample} is the spin polarization of the sample. From this it is possible to extract a map of the spin polarization of the tunneling current for a given relative orientation of tip and sample magnetizations. This is done by subtracting two images with the tip polarized along a parallel and anti-parallel direction, thus canceling the non-magnetic part of the image relating to I_0. An alternative method consists of directly subtracting a non spin polarized image from a spin polarized one recorded on the same surface and measured with the same STM tip, if that is available. We have generated a non spin polarized image by averaging images taken in opposing field directions, thereby canceling the contribution of the spin polarization of the tip. We then use this non-magnetic image to extract the purely magnetic

contrast from data taken in only one field direction. This method produces an equivalent image of the spin polarization along a given direction as subtracting images acquired in field with opposite directions, compare fig. S6.

Section S6. Model for the magnetic structure at *x* = 0.12

A model that reproduces the SP-STM results for the magnetic structure of $\mathrm{Fe_{1.12}Te}$ is given by

$$\begin{aligned} S_a &= \frac{1}{\sqrt{2}}\cos(q_a X) + \frac{1}{\sqrt{2}}\cos(q_b Y) \\ S_b &= \sqrt{2}\cos(q_a X) \\ S_c &= \frac{2}{\sqrt{2}}\cos(q_a X) + \frac{1}{\sqrt{2}}\sin(q_b Y) \end{aligned} \tag{S1}$$

where S_a, S_b and S_c are the components of the local magnetization along the a, b and c lattice directions respectively. X and Y are the position across the sample surface, X being along the a direction and Y being along the b direction.

Section S7. Model for the magnetic structure at *x* = 0.2

To determine the magnetic structure underlying the double-**q** order, we have created simulated STM images for different models of the magnetic order. The order that we find most closely reproduces the SP-STM data is comprised of two spin spirals that propagate through the crystal along the Fe-Fe $[1\bar{1}0]$ direction. The spirals alternate between being right handed and left handed corkscrews on alternate rows of Fe atoms. (See Fig. 6 in main text). Mathematically this order is described by

$$\begin{aligned} S_a &= \frac{1}{\sqrt{2}}\cos(q_a X + \frac{\pi}{4}) + \frac{1}{\sqrt{2}}\cos(q_b Y + \frac{\pi}{4}) \\ S_b &= \frac{1}{2}\cos(q_a X + \frac{\pi}{4}) + \cos(q_b Y + \frac{\pi}{4}) \\ S_c &= \sqrt{2}\cos(q_a X + \frac{\pi}{4}) + \frac{1}{2}\sin(q_b Y + \frac{\pi}{4}) \end{aligned} \tag{S2}$$

The simulated SP-STM images are created by taking the dot product of $[S_a, S_b, S_c]$ with a unit vector in a given direction that represents the magnetization of the tip. The intensity of the magnetic order along a given direction is determined from the height of the corresponding peak in the modulus of the Fourier transformation. The results of this process for a rotation of the tip spin in the plane of the Fe lattice (rotation from a towards b axis) and out of the plane of the Fe lattice (rotation from b towards c axis) are shown in fig. S7. For the ϕ rotation a tip parallel to the crystal a axis is represented by $0°$. For the θ rotation a tip parallel to the crystal b axis is represented by $0°$. This model faithfully reproduces the STM data shown in Fig. 5 of the main text.

Table S1. Crystal structure of $Fe_{1+x}Te$ at different excess iron concentrations x. Values for Ψ shown as grey background in Fig. 6(e) for different excess Fe concentrations x obtained using the lattice parameters in Ref. (*2*).

x	a (Å)	b (Å)	Ψ (%)
0.04	3.83	3.785	1.19
0.05	3.83	3.785	1.19
0.07	3.834	3.785	1.29
0.075	3.834	3.785	1.29
0.12	3.833	3.79	1.16
0.145	3.82	3.79	0.79

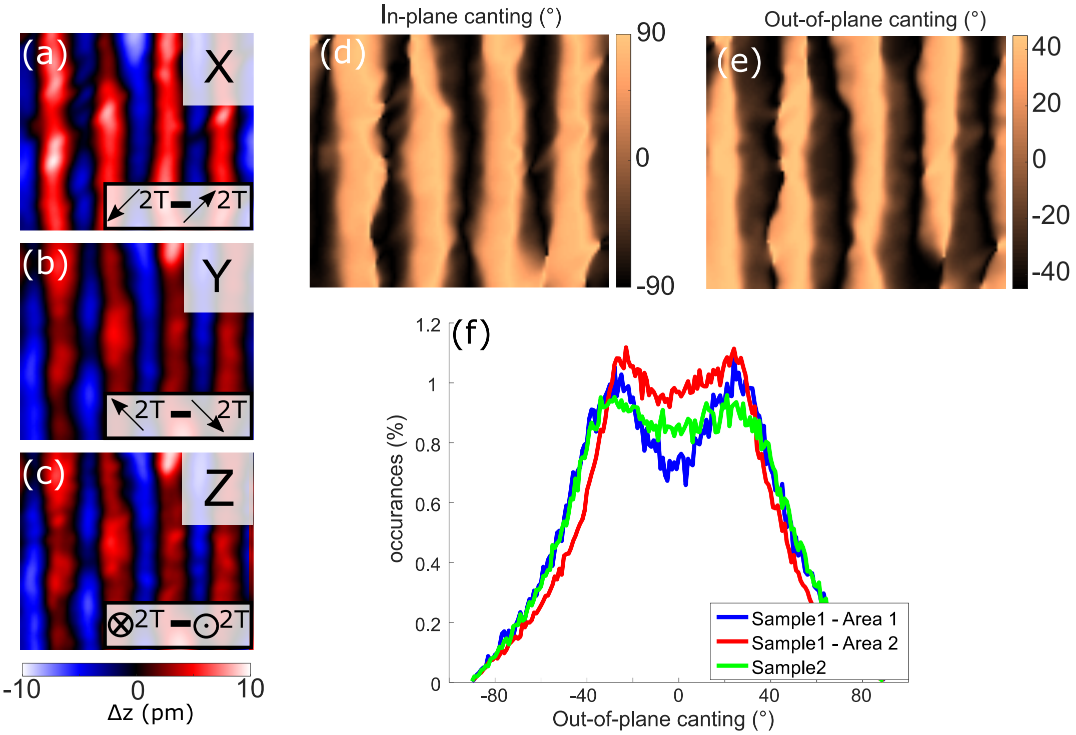

Fig. S1. Spin-polarized imaging at low excess iron concentrations $x < 0.12$. (a-c) SP-STM images of an $\mathrm{Fe_{1.06}Te}$ sample constructed from subtraction of two topographic images taken with the tip polarized along the two opposite directions. (a-b) two in-plane orthogonal directions and (c) out-of-plane direction respectively [$(2.5 \times 3.5)\mathrm{nm}^2$]. (d) The in-plane canting from the a axis determined from (a-c). (e) The out-of-plane canting from the a-b plane determined from (a-c). (f) Histograms of out-of-plane tilting angle for multiple 3D spin polarization studies across multiple samples.

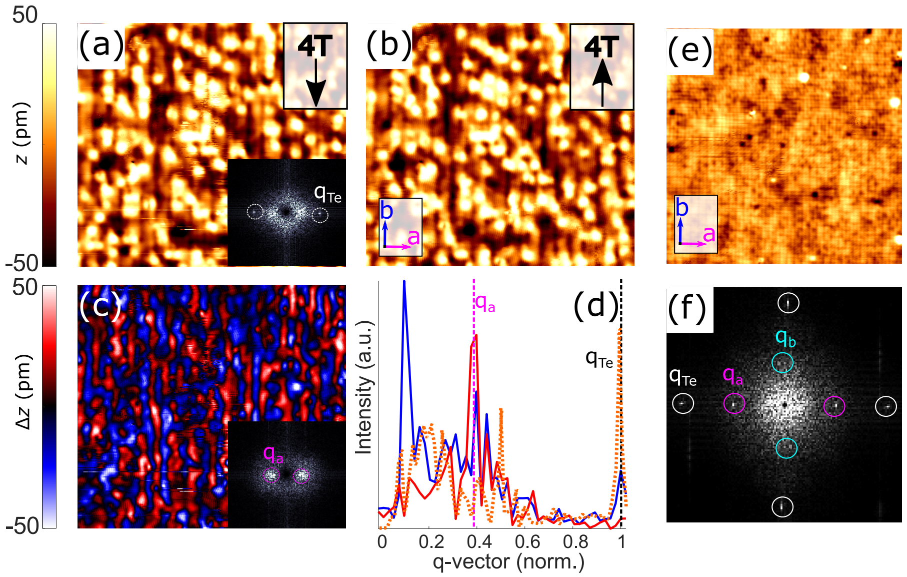

Fig. S2. Spin-polarized imaging at high excess iron concentrations $x > 0.12$. (a) SP-STM image of the $\mathrm{Fe_{1.16}Te}$ surface [$(17.5 \times 17.5\mathrm{nm}^2)$, $I = 0.1\mathrm{nA}$, $V = 50\mathrm{mV}$] with the tip polarized anti-parallel to the crystal b axis. Inset: Fourier transform of (a). (b) as (a) but with field reversed by $180°$. (c) the difference of (a) and (b) showing the magnetic contrast. Inset: Fourier transform of (c). (d) linecuts from the Fourier transforms of (a) and (c). Blue - linecut from (a) showing the Te Bragg peak. Red - linecut from (c) showing the peak due to the incommensurate magnetic order (q_a=0.39) in an area with high surface concentration of excess iron. In addition, a linecut from an area with low surface concentration of excess iron (f) is shown exhibiting the peak at $q = 0.5$ associated with the commensurate order (dashed orange line). (e) SP-STM image of an area on the same sample that has been cleaned of excess Fe [$(23 \times 23\mathrm{nm}^2)$, $I = 0.4\mathrm{nA}$, $V = 200\mathrm{mV}$] showing the checkerboard magnetic order. (f) Fourier transform of the image shown in (e) showing magnetic peaks at the bicollinear vector q_a and with order in the b direction q_b.

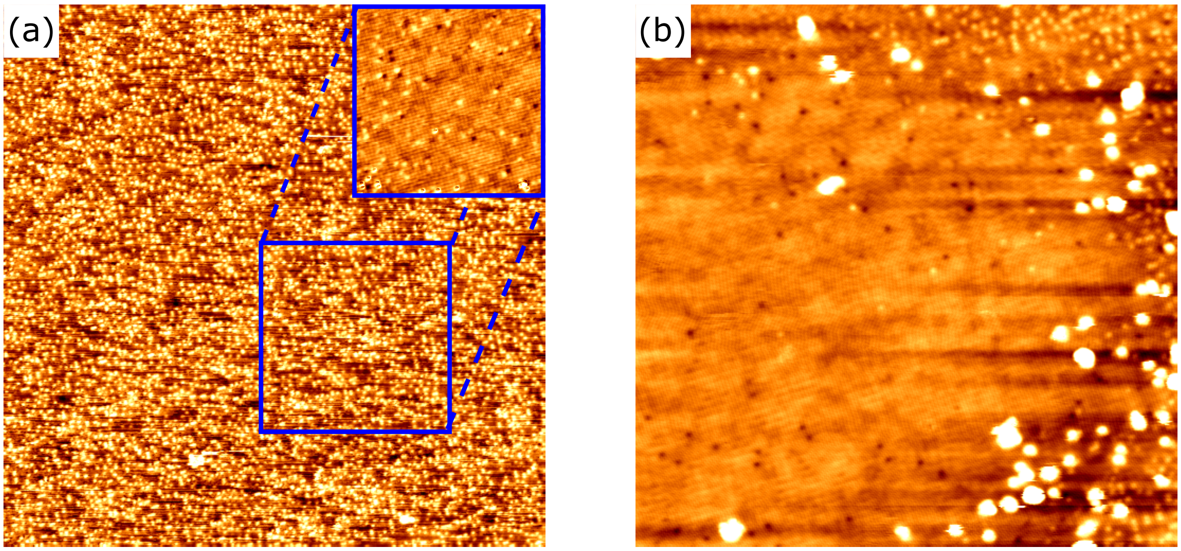

Fig. S3. Manipulation of surface excess iron with aggressive tunneling parameters. (a) SP-STM image of an $\mathrm{Fe}_{1.12}\mathrm{Te}$ area $(80 \times 80)\mathrm{nm}^2$ before any manipulation of the surface interstitial Fe atoms. Inset - Image of the blue highlighted area after aggressive cleaning of the surface has been conducted. The concentration of the interstitial Fe atoms is severely reduced. (b) $(50 \times 50)\mathrm{nm}^2$ SP-STM image showing the boundary between the area after it has been completely cleaned and the neighboring uncleaned substrate. The cleaned area has been stripped completely of excess Fe atoms as can be seen in comparison to the neighboring uncleaned area and the image (a) of the area as it appeared originally.

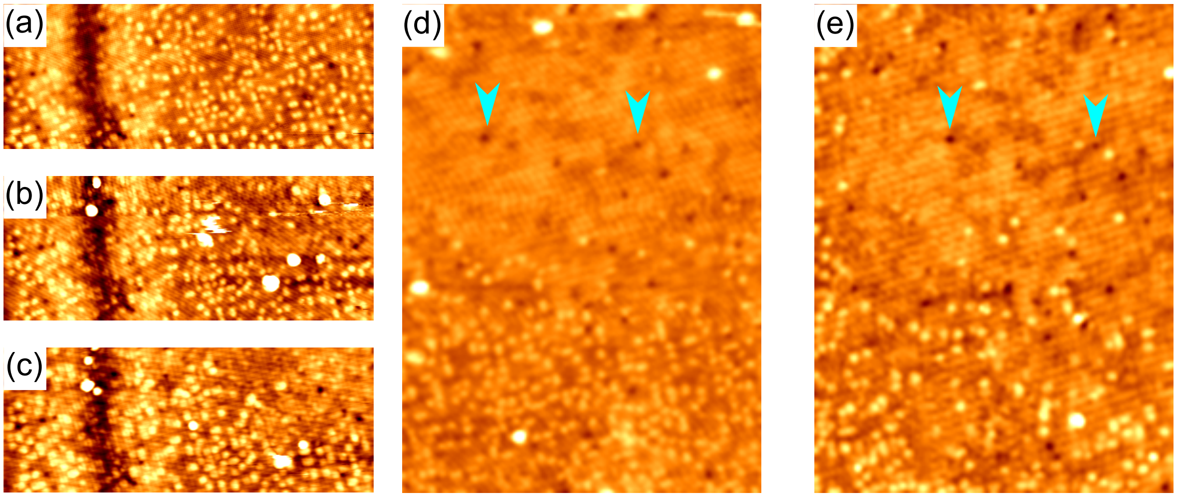

Fig. S4. Manipulation of surface excess iron with moderate tunneling parameters. (a-c) SP-STM images of the same $(34 \times 10)\mathrm{nm}^2$ area on a $\mathrm{Fe}_{1.1}\mathrm{Te}$ sample imaged successively with moderate tunneling parameters ($I = 1\mathrm{nA}$ and $V = 100\mathrm{mV}$) and a slow feedback response time, leading to removal of excess iron. The Fe atoms in the top right corner can be seen to be dislodged from their original positions to form iron clusters, it is then these clusters that are removed by the tip. (d) and (e) the same location on a sample of $\mathrm{Fe}_{1.12}\mathrm{Te}$ at different stages of the cleaning process through scanning with a tunneling current close to $2\mathrm{nA}$ and a bias voltage of $150\mathrm{mV}$, with (d) having been imaged before (e). Both images were taken at intervals during the cleaning process ($V = 150\mathrm{mV}$, $I = 50\mathrm{pA}$). Highlighted the same hole like defects for reference. The excess Fe atoms in the bottom section of the image can be seen to have been dislodged from their original locations and the overall concentration of Fe atoms has been reduced indicating the success of the manipulating process.

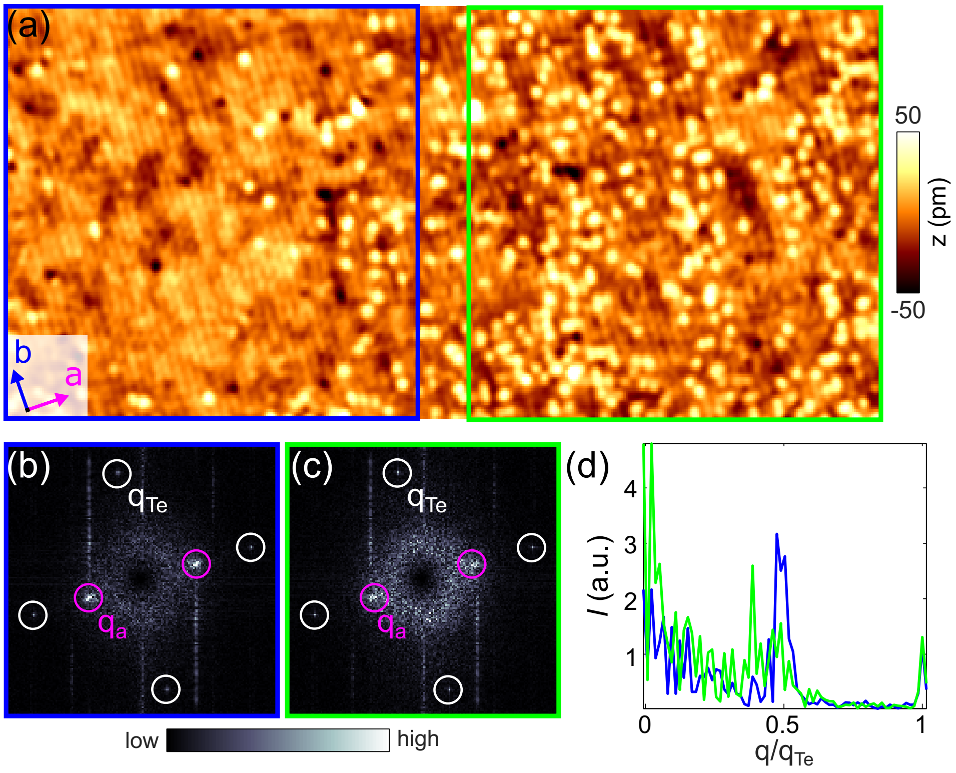

Fig. S5. Manipulating surface magnetic order. (a) SP-STM images as in Fig.3 of the main text. (b) and (c) show the Fourier transformation in the blue and green regions of (a). (d) line cuts through the Fourier transformations shown in (b) and (c), highlighting that the commensurate wave vector is dominant in the blue region, where excess iron has been removed at the surface, and that the magnetic order is dominant at an incommensurate wave vector in the excess iron-rich region.

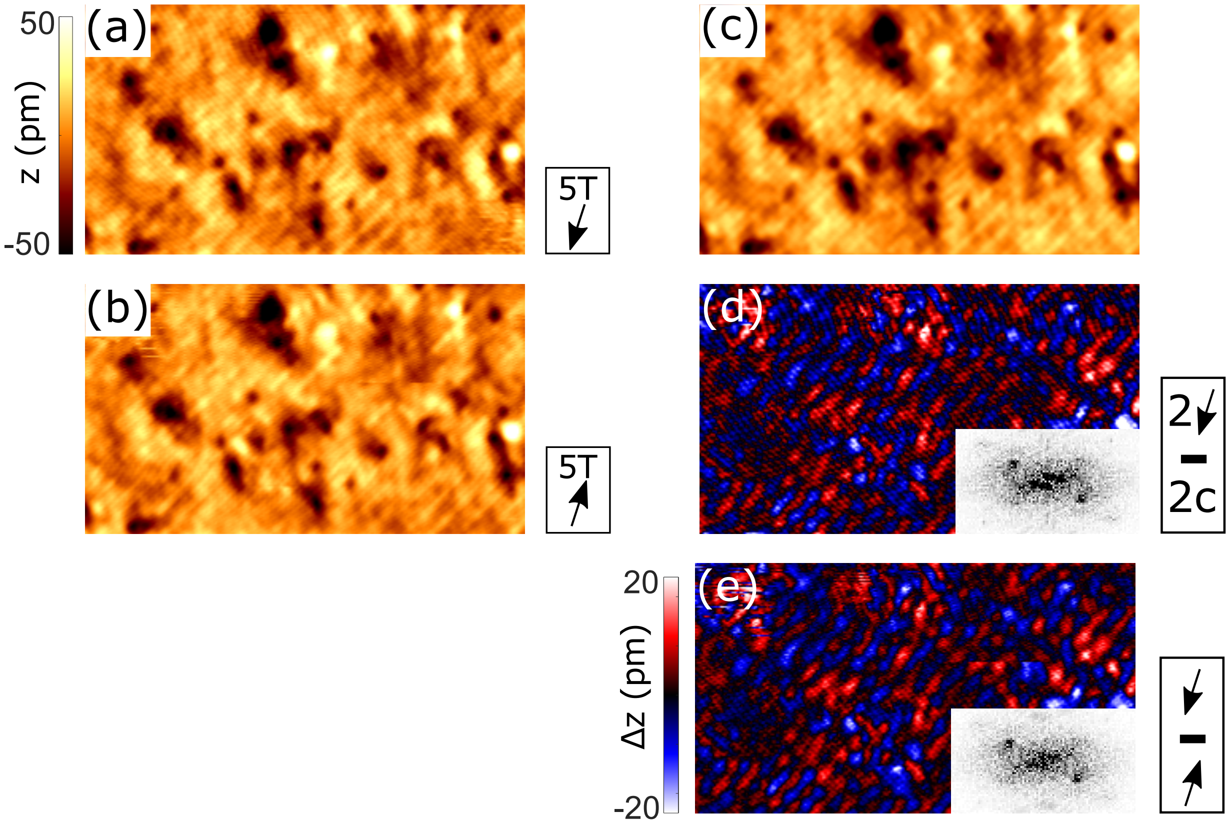

Fig. S6. Extracting surface spin-polarization. (a) Topographic image [$(19 \times 10.8)\mathrm{nm}^2$, $V = -40\mathrm{mV}$ and $I = 0.1\mathrm{nA}$] of an $\mathrm{Fe}_{1.2}\mathrm{Te}$ surface imaged with a ferromagnetic tip polarized with a 5T field along the direction indicated by the arrow. (b) The same area as (a) imaged with the tip polarized along a field rotated by $180°$. (c) The average of 22 images of the same area taken with tips polarized along random field orientations. The images were evenly split with half showing the magnetic order at zero phase and half with the phase of the ordering shifted by π. (d) The difference (2a)-(2c) showing the magnetic contrast obtained by subtracting the averaged image. Inset- Fourier transform of (d). (e) The difference (a)-(b) showing the magnetic polarization of the sample surface along the applied field direction. Inset- Fourier transform of (e) showing magnetic peaks. The equivalence of the two methods of extracting the spin polarization can be seen by the comparatively similar spin polarized images and their Fourier transforms.

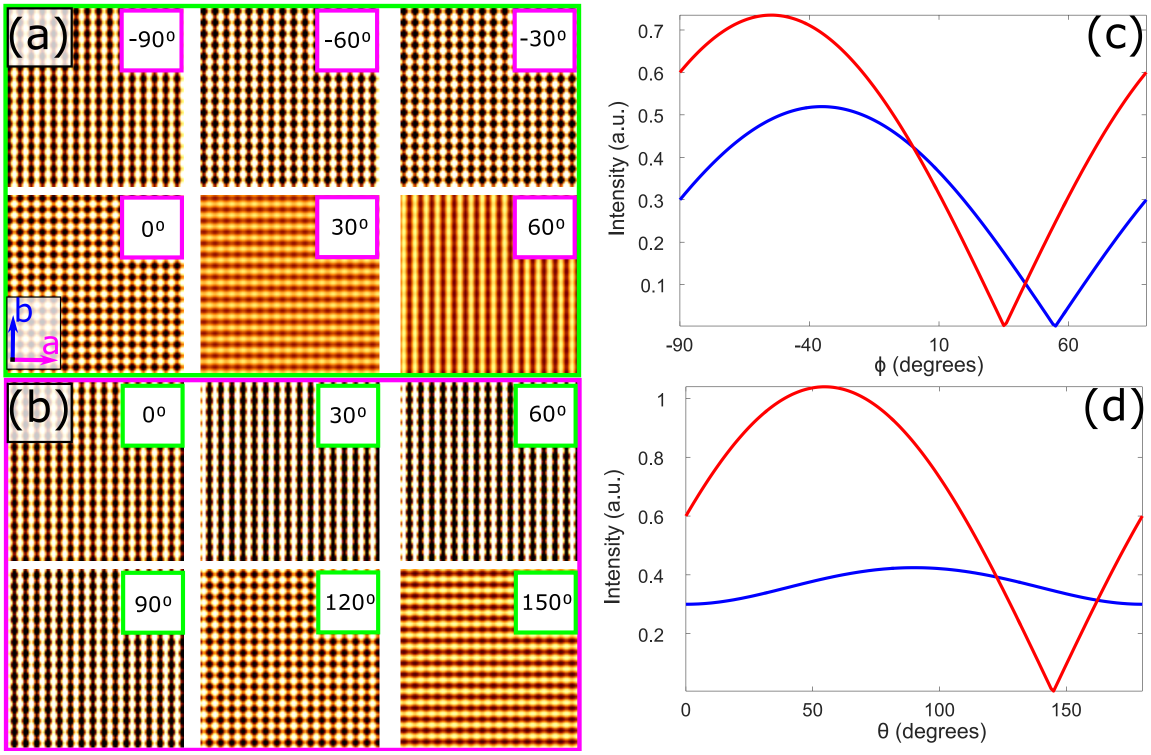

Fig. S7. Simulated SP-STM images for ***x*** **= 0.2.** (a) Images (green box) corresponding to an in-plane rotation of the tip spin through $30°$ steps from being parallel to the b axis at $\phi = -90°$ to being parallel to the a axis at $\phi = 0°$ (color scale between -1 and 1). (b) Images (pink box) corresponding to an out-of-plane rotation of the tip spin from being parallel to the b axis at $\theta = 0°$ to being parallel to the c axis at $\theta = 90°$. (c) Intensities of the magnetic peaks from the Fourier transform of the simulated SP-STM images $I(q_a)$ (red) and $I(q_b)$ (blue) as a function of in-plane angle ϕ of the tip magnetization. (d) Intensities of the magnetic peaks $I(q_a)$ (red) and $I(q_b)$ (blue) as a function of out-of-plane angle θ of the tip magnetization.
